# Medicinal plants used by traditional healers to treat malignancies and other human ailments in Dalle District, Sidama Zone, Ethiopia

**DOI:** 10.1186/s13002-018-0213-z

**Published:** 2018-02-14

**Authors:** Nigatu Tuasha, Beyene Petros, Zemede Asfaw

**Affiliations:** 1 College of Natural and Computational Sciences, Mizan-Tepi University, P.O. Box 260, Tepi, Ethiopia; 2 Department of Microbial, Cellular and Molecular Biology, College of Natural Sciences, Addis Ababa University, P. O. Box 1176, Addis Ababa, Ethiopia; 3 Department of Plant Biology and Biodiversity Management, Addis Ababa University, P. O. Box 3434, Addis Ababa, Ethiopia

**Keywords:** Herbal medicine, Indigenous knowledge, Sidama, Traditional healers

## Abstract

**Background:**

Medicinal plants (MPs) used by traditional healers (THs) were investigated in Megera and adjacent subdistricts (kebeles) of Dalle District, Sidama Zone, southern Ethiopia. The objective of the study was to identify and record MPs and their traditional uses in the treatment of various human ailments with emphasis on malignancies and other most frequently reported diseases.

**Methods:**

Traditional medicinal knowledge held by 20 THs was investigated following standard ethnobotanical approaches. Guided field walk, free listing, rigorous individual interviews with extended discussions, and local market surveys were employed to obtain information. Preference rankings, paired comparisons, use value (UV) index, frequency of citation (FC), fidelity level (FL), and informant consensus factor (ICF) matrices were engaged to identify MPs used to treat malignancies and the other most prevalent human ailments.

**Results:**

Seventy-one MP species belonging to 63 genera and 46 families, used to treat 39 human ailments, were recorded. A high proportion of the species recovered was shrubs (35.2%); while 64.7% were retrieved from the wild habitat. Leaves were the main part of the MPs used (42.9%), followed by fruits/seeds (13%); all preparations were made from fresh materials and about 27.9% involved boiling. The frequent route of delivery was oral (77.9%), followed by dermal (17.6%). About 40.8% of the MPs were used for treating two or more ailments. About 19.7% of the MPs were used to treat malignancies (ICF = 0.86) among which the plant species *Sideroxylon oxyacanthum* was the most frequently used (FL = 70%). The species *Podocarpus falcatus* and *Hagenia abyssinica* were preferred to treat jaundice and deworm in helminthiases, respectively.

**Conclusion:**

The study area is very rich in plant biodiversity, and the herbal medicine is an integral part of the traditional healthcare system. The MPs are exposed to various destructive anthropogenic activities, and this situation calls for integrated conservation measures. Furthermore, the rich ethnomedicinal knowledge held by the Sidama community at large and TM practitioners, in particular, needs an in-depth study and documentation. Investigations of the MPs with high ICF, FL, and UVs to malignancies, jaundice, and helminthiases could possibly contribute to future drug development efforts.

## Background

Ethiopia is known for its ancient civilization and is home to people of diverse ethnolinguistic backgrounds [1]. It stretches from about 120 m below sea level (the Kobat Sink in the Afar depression) to 4543 m above sea level (the highest peak of Ras Dashen mountain) [2, 3]. This feature had its share for the creation of varied ecosystems and a high diversity of vascular plants (about 6027 species) with 10% endemism [4–7]. More than 1000 plant species have so far been reported for use in Ethiopia’s traditional herbal medicine, and about 33 of these species are endemic to the country [8, 9]. Ethiopia is one of the six plant-rich countries of Africa where about 60% of the plants are said to be indigenous and most of them with healing potential [10]. Jansen [11] had once stated that almost all plants of the Ethiopian flora are used medicinally in different parts of the country in one way or the other.

In Ethiopia, over 70% of the people depend on traditional medicines (TMs) for their healthcare, and more than 95% of the preparations are made from plant origin [12, 13]. According to various reports, local people in rural Ethiopia mostly rely on TMs for healthcare services and others revert to TM when modern health services fail due to various reasons [14]. Dissatisfaction with modern medicines and lack of their efficacy, especially in the cases of certain human ailments including cancer, liver diseases, herpes zoster, eczema, swelling, and hemorrhoids, were some of the reasons that traditional healing systems were preferred over conventional medicines [15]. There are case reports of TMs being the preferred treatment options for the early stages of some diseases, including malignancies [16].

In Ethiopia, environmental degradation, deforestation, intermittent drought, and various anthropogenic activities are threatening the natural resources in general, and MPs are most affected. A few ethnobotanical studies undertaken in Sidama Zone, where the present study was conducted, showed that TM is an integral part of the healthcare system of the Sidama people [17–19]. This corroborates the national report on TM use in healthcare [12, 20].

The hypothesis for the present study emerged from observations on the long history of use of herbal medicine to treat human ailments by traditional healers (THs) among the Sidama people. It was deemed necessary to retrieve essential ethnobotanical information from the THs before the MP resources and the associated indigenous knowledge are lost. Therefore, we hypothesized that the THs that provide treatment for various human ailments have valuable knowledge about the traditional MPs used to treat various malignancies, jaundice, and helminthiases. Hence, the objective of the study was to record, compile, and document ethnobotanical knowledge held by THs of Megera subdistrict (kebele) and three other adjacent kebeles of Dalle District, Sidama Zone, southern Ethiopia. Emphasis was given to the MPs used to treat various malignancies, jaundice, and helminthiases.

## Methods

### Description of the study area

**Fig. 1:**
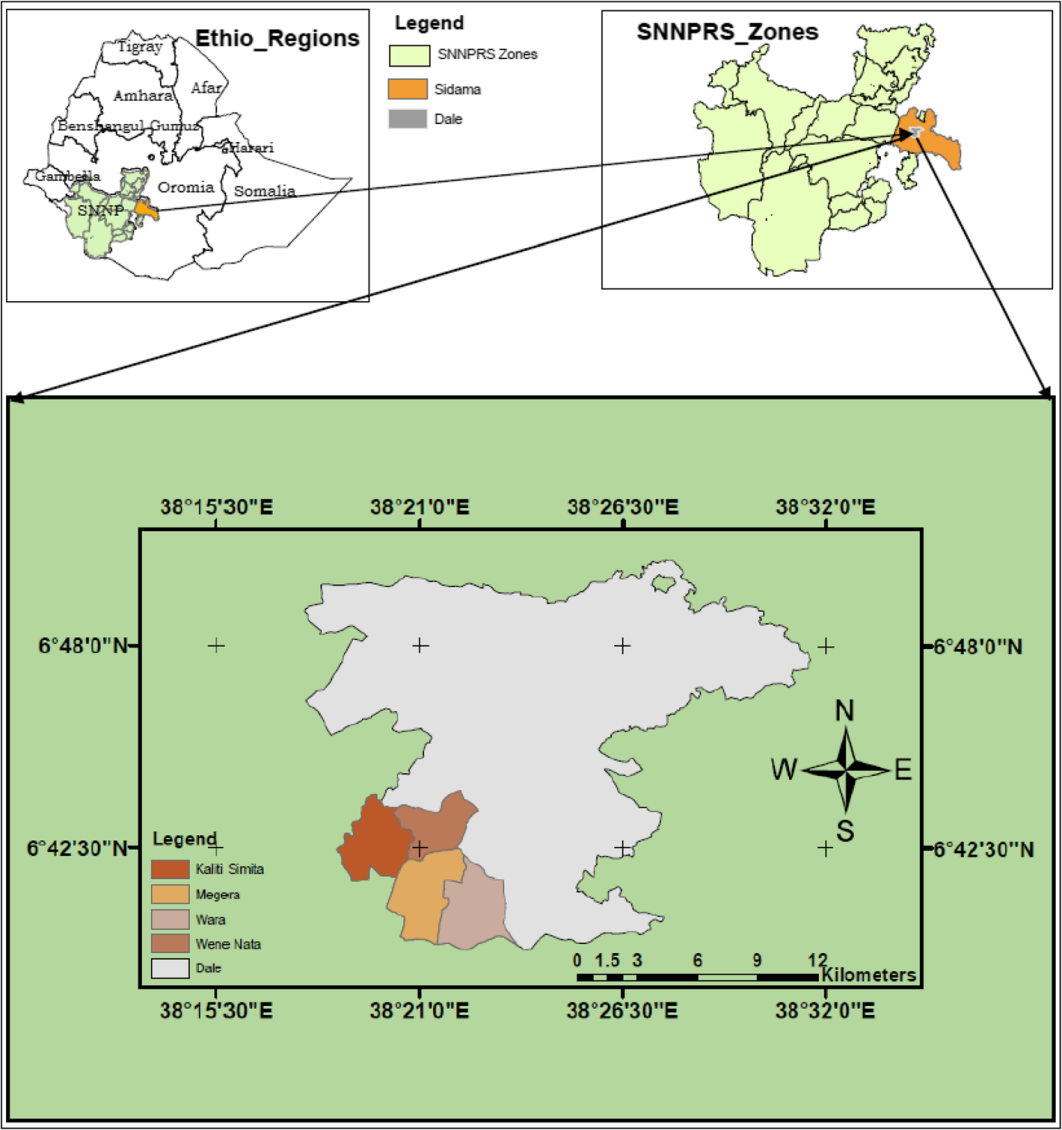
Map showing the study area in Dalle District, Sidama Zone, Ethiopia

The study was conducted in Megera and three adjacent kebeles of Dalle District, one of the 21 districts (19 rural and 2 autonomous administrative towns), of Sidama Zone (Fig. 1). Sidama Zone is located in Southern Nations, Nationalities and Peoples’ Regional State, Ethiopia. The city of Hawassa, located 275 km south of Addis Ababa, is the Regional and Zonal capital. The study area lies about 35 km south of Hawassa. The inhabitants of Dalle District belong mainly to the Sidama nationality and their native language, Sidamu-afoo, belongs to the Cushitic languages family. According to the national census report projected for the year 2017, the zone has a total population of 3,668,304 (50.41% male and 49.59% female) [21]. Dalle District has 35 kebeles and a population of 317,246 (male = 50.31%, female = 49.69%) of which 252,739 (79.67%) dwell in the rural parts of the district [21, 22]. The study area belongs to a moderate (“Wo’richo” or “Woinadega”) climatic zone and lies between the altitudinal ranges of about 1700 and 1850 m above sea level.

### Reconnaissance survey and informant selection

Between the months of February and April 2015, local administrators, elders (*Hayyoole*) (respected/influential personalities), rural development agents, and the center for training farmers were contacted and their recommendations obtained on how to locate and approach the local THs. Twenty THs (14 males and 6 females) were identified from Megera kebele and three other adjacent kebeles (Wene Nata, Wara, and Kaliti Simita) of the district, and their backgrounds were recorded (Table 1). They are all THs, mostly working with herbal medicine to varying extent. Their ages ranged from 38 to 82 years (mean = 62.3; SD = 9.75). The knowledge they held was recorded based on their preferences. Most frequently mentioned human ailments of public health importance around the area were also investigated in association with the symptoms of the diseases as verified by their indigenous knowledge. Ailments of public health importance are diseases/conditions that are a threat to public health and those can be identifiable on an individual or at a community level. The list of ailments was cross-checked with data of ten most prevalent ailments in Dalle District health department [23].

**Table 1:** Demographic data of the traditional healers in Dalle District, Sidama Zone, 2015

Demographic data (*N* = 20)	Frequency (%)
Gender	
Male	14 (70)
Female	6 (30)
Educational status	
No education	11 (55)
Basic reading and writing skills only (adult education)	2 (20)
Elementary school (grade 1 to 6)	6 (30)
Further education (junior secondary and high school education)	1 (5)
Religion	
Cultural religion	6 (30)
Protestant Christianity	9 (45)
Other Christians (e.g., Jehovah Witnesses, Seventh Day Adventists)	5 (25)
Ethnicity	
Sidama	20 (100)
Years of experience as a TH (range)	
< 10 years (least experienced TH has 4 years of experience)	3 (15)
10–20 years	5 (25)
More than 20 years	12 (60)

### Ethnobotanical data collection

Rigorous individual interviews and extended discussions with the practitioners, guided field walk, free listing, and local market surveys were the main data collection methods employed in this study. The interaction with the THs was made carefully paying due attention to Bennett’s Golden Rules for ethnobotany fieldwork [24]. Standard pre-prepared formats were used to record the information provided by the THs about the local name of the MP, the part(s) used, preparation methods, disease(s) treated, the route of delivery, possible side effects, and the antidotes used [25–27]. Plant growth habit, habitat, conservation status, potential threats, and management prospects of the reported MPs were also included in our interview guide. The checklist used was translated into the local language (Sidamu-afoo), and the responses of the THs were recorded in the data collection tool. All interviews and discussions were conducted in Sidamu-afoo by the principal investigator. Each TH was visited and interviewed two to three times to ensure consistency of the information provided. The local market survey was carried out at two comparatively large marketplaces in the study area (*Megera Qawaallanka* and *Wara Dikko*). During the survey, Sidamu-afoo names of the plants, parts used, and the purpose it was sold for were recorded.

### Plant specimen collection and identification

After recording the ethnobotanical information, voucher specimens of the MPs were collected from the wild and home gardens guided by the THs, from July to September 2015. GPS was used to record data on altitude and geographical coordinates. The specimens were numbered and the Sidamu-afoo names of the species were recorded, pressed, and properly dried in the field. Appropriate documentation was made with photographic pictures of the area and the mature individual plant at the site of collection. The identification of specimens was undertaken using the relevant volumes of the Flora of Ethiopia and Eritrea, by comparing with authenticated specimens and finally confirmed by a plant taxonomic experts. Finally, the specimens with labels were deposited at the National Herbarium (ETH), Addis Ababa University.

### Data analysis

Data entry and validation, descriptive statistical techniques of qualitative and quantitative ethnobotanical records, percentages, and frequencies were applied to summarize the data. Furthermore, analytical methods of ethnobotany including preference ranking, paired comparison, informant consensus factor (ICF), fidelity level (FL) index, use value (UV), and frequency of citation (FC) measurements were employed.

Most trusted MPs considered to be effective for the common human illnesses were identified based on the agreement among the THs using the free list data that showed the degree of consensus among them.

Preference ranking was undertaken according to Martin’s procedure [26], using six key informants based on the values of free listing and informant consensus. Accordingly, selected MPs used to treat malignancies, hepatitis (jaundice), and for deworming in helminths were subjected to this procedure. The healers gave MPs believed to be most effective to treat those illnesses the highest value (5), and the least effective was given the lowest value (1). The preferences of the top five MPs said to be used for treating malignancies, jaundice/liver disease, and helminthiases were subjected to a ranking by six THs separately. The practitioners were requested to compare selected MPs based on their knowledge to treat the illnesses. The values given to each species were summed up, and the ranks were determined based on the total score. This procedure helped to identify the MP species that are very likely to be the most effective for treating the specific disease based on the consensus/agreement among the healers.

Paired comparisons were used to evaluate the degree of preference or levels of importance of MPs that had high consensus among the THs. The randomly paired comparison was made according to Martin’s procedure [26] to determine the effectiveness and popularity of the five MP species claimed to be frequently used to treat malignancies in the study area. Five THs taken as key informants were asked to compare the randomly paired and sequenced anticancer MPs. Then, their response on the value of each MP was recorded, and the total score of all key informants on each MP was summed and ranked. Finally, the plant with the highest score was described as the most effective anticancer MP as per healers’ perceptions.

The ICF was applied in the manner used by Heinrich et al. [28], applying the following formula:

$$ \mathrm{ICF}\kern0.5em =\kern0.5em \frac{Nur- Ns}{Nur-1} $$

where *Nur* is the number of use reports (use citations), and Ns is the number of species used for each citation by TM practitioners. If a TM practitioner mentioned a plant used for the treatment of one ailment (e.g., cancer) or for more than one ailment, then it is considered as one use report in line with recommendations from other sources [29]. Values ranging between 0 and 1 are obtained, where a value close to one (1) is obtained when only one or few species are reported by the majority of TM practitioners. Conversely, low ICF values (close to zero) show disagreement among the TM practitioners, which may include reasons like having different experiences, keeping information with strict secrecy, or lack of prior information exchange among them [30].

The ratio of informants who independently reported their use of a species for the same major purpose to the total number of informants who mentioned the plant for any use is described as FL [31]. FL was calculated for the most frequently reported diseases/ailments as:

$$ \mathrm{FL}\kern0.5em \left(\%\right)\kern0.5em =\kern0.5em \frac{Np}{N}\times 100 $$

where *Np* is the number of informants that claim the use of a plant species to treat a particular disease, and *N* is the number of informants that use the plant as a medicine to treat any given disease [31]. The medicinal plant use diversity was calculated by adding the number of uses the healers listed for each species.

The use value (UV) index helped to evaluate the relative importance of each medicinal plant species based on its relative use among informants. Based on the citation of plants during interview sessions, UVs were calculated following the approach given by Prance [32] and Andrade-Cetto and Heinrich [33] using the formula:

$$ \mathrm{UV}\mathrm{s}\kern0.5em =\kern0.5em \left(\sum \mathrm{UV} is\right)/(ni) $$

where ∑UV*is* is the sum of the total number of use citations for a given species by all informants;

n*i* is the total number of informants interviewed for species s.

The frequency of citation (FC) was also computed to determine the percentage of respondents who have knowledge regarding the use of a species as follows:

$$ \mathrm{FC}\kern0.5em =\kern0.5em \left(\sum \mathrm{UV} is\right)/(ni)\kern0.5em \times \kern0.5em 100 $$

where ∑UV*is* is the sum of the total number of use citations for a given species by all informants;

n*i* is the total number of informants interviewed for species s.

## Results

### MPs reported by THs

The study recorded 71 species (69 angiosperms and 2 gymnosperms) of MPs distributed in 63 genera and 46 families (Table 2). Thirteen families were represented by two or more species of MPs, whereas 33 families were represented by only one species each. The family Lamiaceae was represented by six MPs, whereas the Asteraceae, Euphorbiaceae, and Rubiaceae were represented by four species each (Table 3). The MPs collected from the study area constituted shrubs that accounted for 35.2%, followed by herbs (26.7%), trees (23.5%), and climbers (14.7%). The majority (64.7%) were sourced from the wild environments while home gardens accounted for 35.3% of the MPs.

**Table 2:** Description on the habit, habitat, parts used, use value, frequency of citation, mode of preparations, route of delivery and human ailments treated by the THs in Megera and adjacent Kebeles of Dalle District, 2015

Family	Scientific name	Vernacular name (Sidamu-afoo)	Habitat	Habit	PU	RA	UV	FC	Preparation and application	Illness/disease condition treated	Collection no.
Acanthaceae	*Hypoestes forskaolii* (Vahl) R. Br.	Ciikkicho	W	Sb	LRt	Or	0.15	15.0	The leaf is pounded, macerated, and drunk; The root is chewed, and the output is swallowed	Helminthiases Severe stomachache	NT056
Alliaceae	*Allium sativum* L.	Waajjo tuma	HG	H	Bu	Or	0.40	40.0	The garlic is peeled, chewed, and swallowed	Tonsillitis Acute severe stomachache	NT054
Aloaceae	*Aloe* sp.	Argiisa	W	Sb	Lf	Or	0.60	60.0	Fresh *Aloe* sp. leaf is chopped, pounded, and small quantity (about 1 teaspoon) is drunk immediately or licked and swallowed	Acute febrile illness (*‘lamootta’/‘dingetegna*’)	NT032
Anacardiaceae	*Mangifera indica* L.	Mango	HG	T	Sh	De	0.20	20.0	The fluid which oozes out while the shoot is cut is applied to the wounded part of the body	Blood clotting and wound healing	NT053
Apiaceae	*Foeniculum vulgare* Mill.	Malkata	HG	H	LSt	Or	0.05	5.0	Pounded, macerated, and are given orally	Blood pressure	NT015
Apocynaceae	*Acokanthera schimperi* (A. DC.) Schweinf.	Qaraaro	W	T	Fr	Or	0.20	20.0	The ripe fruit (only the sweet one) is pounded, macerated, and mixed with water, and the patient drinks about 1 and ½ cup of the preparation in the morning before breakfast	A syndrome called *‘Arrisho*’ *‘Usso/yebird beshita*’ (a stabbing pain)	NT018
Asparagaceae	*Asparagus africanus* Lam.	Buticho	W	Sb	Rt	Or	0.30	30.0	The root is pounded, boiled, and drunk	*‘Usso/yebird beshita*’ (a stabbing pain), treated early in the morning before breakfast; Febrile malaria (this time, but not necessarily in the morning)	NT026
Asteraceae	*Acmella caulirhza* Del.	Bexxo	HG	H	Fl	Or	0.45	45.0	Chewed and swallowed	Tonsillitis	NT004
	*Lactuca inermis* Forssk.	Ameessa	W	Cl	Lf	Or	0.25	25.0	The leaf is chopped, boiled, and the filtrate is drunk	*‘Lagote dhibba*’ (growth retardation)	NT064
	*Vernonia amygdalina* Del.	Hechcho	W	Sb	Lf	Or	0.55	55.0	The leaf of *Vernonia amygdalina* is pounded, macerated, and 1 cup of the preparation is given orally	Febrile malaria and helminthiases	NT043
	*Vernonia auriculifera* Hiern.	Reejjicho	W	Sb	Sh	De	0.60	60.0	The leaf from the shoot of the plant, often with *Solanum dasyphyllum,* is gently rubbed and applied on the wounded surface	Wound healing	NT057
Boraginaceae	*Cordia africana* L.	Waaddicho	W	T	BFr	Or	0.15	15.0	The bark is pounded, boiled, and drunk; or the ripe fruits are boiled, and the sweet juice is drunk for 1 week (7 days)	Gastritis	NT041
Caricaceae	*Carica papaya* L.	Paappaye	HG	Sb	LFr	Or	0.30	30.0	The fresh leaf of *Carica papaya* is home dried, pounded, boiled, and mixed with sugar is taken orally for some time (often mixed with fresh leaves of *Ajuga integrifolia*); Fruit of *Carica papaya* with the fruit of *Zanthoxylum chalybeum* are pounded, macerated, filtered, and drunk	Blood pressure Gastritis	NT044
Celesteraceae	*Maytenus undata* (Thunb.) Blakelock	Kincho	W	Sb	Br	Or	0.15	15.0	The bark is pounded, boiled, and filtered, and 1 cup is drenched	Stomachache in infants	NT021
Clusiaceae	*Garcinia buchananii* Baker	Soloolsa	HG	T	Br	Or	0.10	10.0	The bark is peeled carefully, boiled, cooled, and drunk; The same application in the third day from the treatment regimen by *Sida ovata*	Sexual impotence in male A syndrome called *‘Arrisho*’	NT036
Commelinaceae	*Commelina benghalensis* L.	Laaluunxe	HG	Cl	Lt	De	0.60	60.0	The latex which oozes out is applied on the affected area	*‘Baararre*’ and *‘ishiisha*’ (fungal skin infections)	NT047
Crassulaceae	*Kalanchoe petitiana* A. Rich	Hanculuulle	W	H	Lf	Oc	0.05	5.0	Oozing fluid of the leaf is spilled into the eyes	Inflammation of an eye due to poisonous plant materials	NT042
Cucurbitaceae	*Cyclantheropsis parviflora* (Cogn.) Harms	Basu baaqula	HG	Cl	FrS	Or	0.10	10.0	The fleshy fruit is boiled and eaten The seeds are roasted and eaten	Constipation; GIT (gastrointestinal tract) discomforts	NT062
	*Peponium vogelii* (Hook.f.) Engl.	Surupha	W	Cl	Fr	Or	0.35	35.0	The juicy ripe fruit (about 2 or 3) are eaten for few days	Febrile malaria, gonorrhea, and *‘magarto*’ (jaundice)	NT010
Cuppressaceae	*Juniperus procera* Hochst ex. Engl.	Hoomme	W	T	LSh	Or	0.30	30.0	The leaf is pounded, and the juice is given orally The shoot is pounded, decocted, and drunk	Liver disease *‘Buutaame*’ (painful swelling)	NT049
Dioscoreaceae	*Dioscorea alata* L.	Bohe	HG	Cl	LTb	De Or	0.30	30.0	The leaf is rubbed gently and applied on the affected part of the skin; The tuber is boiled and eaten for a certain amount of time	*‘Baararre*’ Diabetes	NT003
	*Dioscorea bulbifera* L.	Kotte Bohe	HG	Cl	Tb	Or	0.05	5.0	Boiled and eaten regularly in small amounts every day; or prepared in the form of soup and drunk	Diabetes	NT035
Euphorbiaceae	*Clutia abyssinica* (Jaub. & Spach.)	Maalaasincho	W	Sb	Lf	Or	0.05	5.0	The leaf of the plant and fresh *Aloe* sp. are mixed, pounded, cold macerated, and given orally	Meningitis	NT007
	*Croton macrostachyus* Del.	Masincho	W	T	Sh	Or	0.70	70.0	The shoot part is boiled and drunk	Febrile malaria, (especially *P. vivax*)	NT011
	*Euphorbia schimperiana* Hochst Ex, A. Rich	Binjille	W	H	Lf	De	0.05	5.0	Mixed with the leaf of *Gouania longispicata* is pounded and creamed on the affected part of the skin	*‘Dhiiga*’ (leprosy)	NT025
	*Ricinus communis* L.	Qombo”o	W	Sb	Rt	Or	0.20	20.0	Fresh root is chewed and swallowed	Breast cancer	NT071
Fabaceae	*Millettia ferruginea* (Hochst.) Bak.	Hengeddicho	W	Sb	Br	Or	0.30	30.0	The bark is washed, pounded, filtered, and given orally	Cancer	NT069
Flacourtiaceae	*Dovyalis abyssinica* (A. Rich.) Warb.	Shiilo	W	Sb	Br	Or	0.65	65.0	The raw bark is chewed and swallowed	*‘Muje*’ (a tumor)	NT017
Icacinaceae	*Apodytes dimidiata* E. Mey. ex Arn.	Doongicho	W	Sb	Br	Or	0.25	25.0	The bark of the plant is boiled, and about 1 cup is given orally	Stomachache *‘Lagote dhibba*’ (retarded growth)	NT008
Lamiaceae	*Ajuga integrifolia* Buch.-Ham. (bugleweed)	Anamuro	HG	H	Lf	Or	0.15	15.0	Fresh leaf is pounded, decocted, and drunk with tea or coffee	Blood pressure	NT046
	*Clerodendrum myricoides* (Hochst.) Vatke	Ma’niisa	W	H	LfR	Or	0.20	20.0	The leaf part is pounded, mixed with honey, and drunk; or its root is boiled, often mixed with the shoot of *Zanthoxylum chalybeum*, is given orally	*‘Naqarsu dhibba*’ (leukemia)	NT006
	*Ocimum lamiifolium* Hochst. ex Benth.	Michete dhagicho	W	Sb	Lf	Na Or	1.00	100.0	The soft leaves are rubbed gently to squeeze a characteristically reddish fluid, collect on a cup, and taken through nostrils very carefully; It may also be drunk with coffee	*‘Miche*’ (an acute viral infection)	NT048
	*Plectranthus garckeanus* (Vatke) J.K. Morton	Toontoona	W	H	Lf	Or De	0.25	25.0	The leaf is boiled, filtered, and the liquid is given orally Raw leaf is put between the fingers of legs	Helminthiases Athletes’ foot (antifungal)	NT034
	*Plectranthus punctatus* (L. f.) L’Hér.	Hellee	HG	H	LSh	De	0.75	75.0	The leaf and shoot are gently rubbed and creamed on the wound	Wound healing	NT031
	*Salvia nilotica* Juss. ex Jacq.	Michete dhagicho (Damakase)	W	Sb	Lf	Na	1.00	100.0	The soft leaves are rubbed gently to squeeze a characteristically reddish fluid, collect on a cup and taken through nostrils very carefully; it may also be drunk with coffee	*‘Miche*’	NT070
Loganiaceae	*Buddleja polystachya* Fresen.	Bullaancho	W	Sb	Lf	Or	0.15	15.0	The leaves are pounded, macerated, and given orally	Cancer	NT013
Malvaceae	*Sida ovata* Forssk.	Qirqixxe	W	Sb	Lf	Or	0.05	5.0	The leaf of *Sida ovate*, often mixed with the root of go’ra (*Rubus apetalus*) is boiled at night, and the cooled preparation is drunk in the morning	*‘A* *rrisho*	NT060
Meliaceae	*Ekebergia capensis* Sparrm.	Goddiicho	W	Sb	Fr	Or	0.25	25.0	The fruits are pounded, filtered, and drunk Few fruits are chewed and swallowed	Cancer Stomachache	NT023
Melianthaceae	*Bersama abyssinica* Fresen.	Xeweerrakko	W	T	Br	Or	0.45	45.0	The bark is pounded, boiled, and a small amount of the preparation is drunk	*‘Naqarsu dhibba*’ (cancer)	NT063
Menispermaceae	*Stephania abyssinica* Dillon and A.Rich.	Kalaala	W	Cl	Lf	Or	0.25	25.0	The leaf part is boiled, and about 1 cup is drunk	Jaundice (liver disease)	NT050
Musaceae	*Ensete ventricosum* (Welw*.*) Cheesman)	Weese	HG	H	RSt	Or De	0.15	15.0	Unfermented newly processed “kocho” is baked and eaten The dry “xusho” (thread) is burned to give “diqillo” (soot), mixed with any kind of body lotion or butter is creamed on the body	Amoebiasis *‘Bijaajo*’/*‘ikek*’ (bacterial skin infection)	NT067
	*Musa x paradisiaca* L.	Muuze	HG	H	St	De	0.20	20.0	The oozing fluid from the stem is applied on a fresh wound	Coagulation and protecting a secondary infection	NT065
Myrsinaceae	*Myrsine melanophloeos* (L.) R. Br.	Morocho	W	Sb	Lf	Or	0.20	20.0	The leaf (often mixed with *Olea capensis*) is pounded, cold macerated, and drunk	Cancer (leukemia)	NT020
Myrtaceae	*Syzygium guineense* (Wild.) DC.	Duuwancho	W	T	BFr	Or	0.15	15.0	The bark is pounded, macerated, and drunk The ripe fruits of the plant are eaten in small amounts for some time	*‘Arrisho*’ (it cures after inducing diarrhea Obesity	NT061
Oleaceae	*Olea capensis* L. f.	Seettaame	W	T	Sh	Or	0.55	55.0	The shoot (often with shoot of *Zanthoxylum chalybeum* and *Clerodendrum myricoides*) is boiled, mixed with honey, and drunk	Cancer	NT022
	*Olea europaea* subsp. *cuspidata* (Wall. ex G. Don) Cif.	Ejersa	W	T	Sh	Oc	0.10	10.0	The soft shoot of the plant is pounded with the addition of small amount of water, filtered, and the pure drop is added at the margins of the cornea	*‘Burdicho*’ (an early stage trachoma)	NT066
Oliniaceae	*Olinia rochetiana* A. Juss.	Noole	W	T	Lf	Na	0.30	30.0	The leaf is heated slightly, rubbed by the hands, and then inhaled through nostrils	Viral common cold	NT009
Passifloraceae	*Passiflora edulis* Sims	Hoophe	HG	Cl	Fr	Or	0.05	5.0	Two ripe fruits are eaten every morning for about 6 months	Blood pressure	NT029
Phytolaccaceae	*Phytolacca dodecandra* L’Herit.	Haraanjicha	W	Sb	Lf	Or	0.25	25.0	Pounded, boiled, and taken orally early in the morning	Helminthiases; as a laxative	NT059
Poaceae	*Cymbopogon citratus* (Hook. & Arn.) Stapf.	Hixicho	HG	H	Lf	Or	0.40	40.0	The grassy leaf is boiled, macerated, cooled, and given to infants orally (esp. drenching)	GIT disorder Boost immunity of breastfeeding infants	NT058
Podocarpaceae	*Podocarpus falcatus* (Thunb.) Mirb.	Dagucho	W	T	SLt	Or	0.65	65.0	The shoot is boiled, and 1 cup is drunk; Oozing liquid from the stem is mixed with cold water and is drunk	*‘magarto*’ (jaundice) Gastritis and amoeba	NT040
Polygonaceae	*Rumex abyssinicus* Jacq.	Shiishoone	W	H	Rt	Or	0.85	85.0	Fresh roots are pounded, boiled, and about 1 cup is given orally	*‘magarto*’ (jaundice)	NT001
	*Rumex nervosus* Vahl	Taare	HG	H	Rt	Or	0.25	25.0	The root is washed and eaten raw The root is pounded, boiled, and given orally	Intestinal parasites *‘Lammootta*’ (acute febrile illness)	NT039
Ranunculaceae	*Clematis simensis* Fresen.	Fiide	W	Cl	LSt	Or	0.35	35.0	The thread is chewed, and the small amount is swallowed; Leaf of the plant is macerated and drunk	Toothache; Cancer	NT037
Rhamnaceae	*Gouania longispicata* Engl.	Daanikuukke	W	Sb	Lf	De	0.10	10.0	Used often mixed with the leaves of *Euphorbia schimperiana*, are pounded and creamed on the affected part	*‘Dhiiga*’ (leprosy and leukoderma)	NT024
	*Rhamnus prinoides* L’Herit.	Xaddo	W	Sb	Lf	Or	0.05	5.0	The leaf, often with the root of *Rubus apetalus*, is boiled, decocted, and drunk before meal	Sexually transmitted diseases (STDs)	NT027
Rosaceae	*Hagenia abyssinica* (Bruce) J.F. Gmel	So”ichote dhagga (qaanqo)	W	Sb	Fr	Or	0.80	80.0	The ripe fruit is pounded, decocted, and 1 cup of the preparation is drunk in the morning before breakfast	*‘So*”*icho*’ (tapeworm)	NT055
	*Prunus persica* (L.) Batsch	Kooke	HG	T	Lf	Or	0.25	25.0	The leaf is pounded, macerated, and drunk	*‘Lamootta*’ (acute febrile illness)	NT033
	*Rubus apetalus* Poir.	Go’ra	HG	Sb	Rt	Or	0.15	15.0	The root is pounded, boiled, and drunk	Tuberculosis and *‘siimmaxo*’ (a disease condition characterized by painful urination like gonorrhea)	NT028
Rubiaceae	*Coffea arabica* L.	Buna	HG	T	Fr	Or	0.10	10.0	The fruit is roasted, pounded, and mixed with salt and honey and is eaten	Severe, frequent, and watery diarrhea	NT051
	*Gardenia ternifolia* Schumach & Thonn.	Gaambeella	W	Sb	LLt	Or	0.15	15.0	The latex is creamed on the tongues of infants; or the leafs are boiled and given orally after cooling	*‘Lagote dhibba*’ (growth retardation)	NT038
	*Pentas lanceolata* Forssk.	Baalaamu dhagga	W	H	Rt	Or De	0.30	30.0	Root is pounded, mixed with water, filtered, and drunk The root is pounded, mixed with small water, and applied on the wound	Cancer Wounds	NT052
	*Rubia cordifolia* L.	Haarre	HG	Cl	Rt	De	0.25	25.0	A TM practitioner chews the root, mixed with salt, and spits on the swelling while uttering the word “fincami” or “dhoohi!”	*‘Buutaame*’ (painful illness with fever and swelling)	NT002
Rutaceae	*Ruta chalepensis* L.	Xenaddaame	HG	H	Sh	Or	0.35	35.0	The shoot is chewed and swallowed	Detoxify poison Painkiller for various ailments including stomachache	NT030
Rutaceae	*Zanthoxylum chalybeum* Engl.	Gadda	W	T	LSh	Or	0.50	50.0	The leaf/shoot (often with the shoot of *Olea capensis* and *Clerodendrum myricoides*) is boiled, mixed with honey, and drunk The leaf of the plant is pounded, macerated, mixed with honey and is given orally	Cancer Gastritis	NT012
Sapotaceae	*Sideroxylon oxyacanthum* Baill.	Bunguude	W	Sb	Lf	Or	0.75	75.0	The leaf part, often mixed with leaf of *Zanthoxylum chalybeum* and honey, is macerated and given orally	Cancer	NT014
Solanaceae	*Solanum aculeatissimum* Jacq.	Haanja Borbodho	W	Sb	LSh	Or De	0.40	40.0	The leaf is chopped with red onion, boiled, and the soup is drunk; The shoot is rubbed gently, and the liquid output is applied on the wounded area	Asthma Wound healing	NT016
	*Solanum dasyphyllum* Schum. & Thonn.	Borbodho	W	Sb	Sh	De	0.35	35.0	The soft shoot of *Solanum dasyphyllum* is gently rubbed, and the liquid output is applied on a wound	Wound healing	NT068
	*Solanum nigrum* L.	Xu’naayye	HG	H	WP	Or	0.25	25.0	The herb is boiled and eaten regularly for about 3 days	*‘Bisu shekkeere*’ (*P. vivax)*	NT005
Tiliaceae	*Grewia flavescens* Juss.	Shishsho	W	T	Lf	Or	0.05	5.0	The leaf is decocted and given orally	*‘Lagote dhibba*’ (growth retardation)	NT019
Zingiberaceae	*Zingiber officinale* Rosc.	Jaanjiweelo	HG	H	RRz	Or Oc	0.15	15.0	The rhizoid is pounded, dried, and mixed with feed/drink The root of *Zingiber officinale* is washed well, gently rubbed with hands, mixed with small amount of water, and dropped into an eye	Pain management for cancer patients *‘Burdicho*’ (early stage trachoma)	NT045

Sidamu-afoo names of illnesses/disease conditions are written in small caps, italic, font 10, within single inverted commas throughout the document

*Abbreviations*: habit (*T* tree, *Sb* shrub, *H* herb, and *Cl* climber); habitat (*W* wild and *HG* home garden), *PU* part used (*Br* bark, *Bu* bulb, *BFr* bark and fruit, *Fl* flower, *Fr* fruit, *FrS* fruit and seed, *Lt* latex, *Lf* leaf, *LFr* leaf and fruit, *LLt* leaf and latex, *LSt* leaf and stem, *LRt* leaf and root, *LSh* leaf and shoot, *LTb* leaf and tuber, *Rt* root, *RSt* root and stem, *RRz* root and rhizoid, *Rz* rhizoid, *Sd* seed, *Sh* shoot, *SLt* shoot and latex, *St* stem, *Tb* tuber, *WP* whole part), *RA* route of application (*De* dermal, *Na* nasal, *Oc* ocular, and *Or* oral), *FC* frequency of citation, *UV* use value

**Table 3:** Taxonomic diversity of the MPs of the study area

Family	No. of genera	% of genera	No. of species	% of species
Lamiaceae	5	7.9	6	8.5
Asteraceae	3	4.8	4	5.6
Euphorbiaceae	4	6.3	4	5.6
Rubiaceae	4	6.3	4	5.6
Rosaceae	3	4.8	3	4.2
Solanaceae	3	4.8	3	4.2
Other 40 families	41	65.1	47	66.2

Some plants reported from the area are generally considered by the healers as broad-spectrum TMs for various human illnesses and often are not specific to certain type of disease(s). These plants include *Allium sativum*, *Ruta chalepensis*, *Zingiber officinale*, *loome* (*Citrus aurantifolia*) and *kishee*/*kokkoso* (epiphytes^1^) (e.g., the basket fern, *Drynaria volkensii*) that grows on various families of seed plants such as *Citrus aurantifolia*, *Coffea arabica*, *Croton macrostachyus*, *Podocarpus falcatus*, and *Olea europaea* ssp*. cuspidata.* According to the THs, from dioecious flowering plant groups, flower and fruit-bearing forms (i.e., with female reproductive structures) are recommended for traditional medicinal use. The opposite ones (plants with male reproductive structures) are considered poisonous or toxic and are not considered for remedy preparation.

### Diseases treated by the THs

It was recorded that more than 39 different human sicknesses were treated by the THs in the study area using remedies from MPs. The most widespread disease conditions in humans, according to the informants, include various forms of malignancies,^2^ jaundice (especially in children), helminthiases, bacterial infections, malaria, chronic obstructive pulmonary disease (e.g., asthma and *Usso*—a stabbing chest pain), diarrhea, swelling, common cold, febrile illnesses (*‘lammootta*’ or *‘dingetegna*’), toothache, gastritis, diabetes, various wounds, and unspecified headaches. A considerable proportion of MPs was found to be used to treat various forms of malignancies (19.7%), non-infectious diseases (e.g., diabetes, blood pressure, asthma, and gastritis) (13.2%), and helminthiases (8.5%).

As explained by the THs, some disease conditions are understood, defined, and expressed in a unique way. The indigenous nature of the medical system in the area is evident from local terminologies applied to the diseases/illnesses and health status of people described by the THs and the community at large. For example, an acute ailment characterized by pain of the hip, irritation during urination, sweating, and loss of appetite is referred to as *‘arrisho’*. A painful illness with fever and swelling on any part of the body, especially on the legs, is known as *‘Buutaame’.* During treating this illness traditionally, the TM practitioner usually utters the words either *fincami*! (to mean “disappear”/“be vanished”) or *dhoohi* (to mean “burst out”) while deeply looking at the swelling. *‘Lammootta’* is an ailment characterized by acute febrile illness and sudden headache that could kill within 2 days if left untreated. Likewise, *‘usso’* is a characteristically severe disease condition with stabbing pain, which is accompanied by sweating and coughing.

### Parts of the MPs used by healers for treating patients

**Fig. 2:**
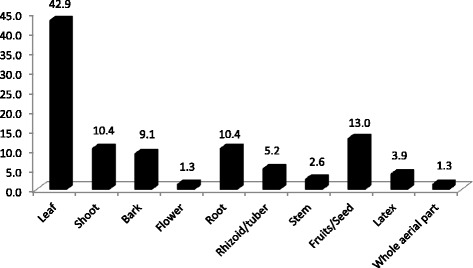
Parts of MPs used by the TM practitioners of Megera and adjacent kebeles to treat various human illnesses (%)

The study revealed that various parts of plants singly or in combinations were used to treat specific ailments in the area (Fig. 2 and Table 2), as explained by THs. Leaves were the most widely used part of MPs with 42.9% usage followed by fruits/seeds (13%). Flowers and whole aerial parts of MPs (1.3% each) were the least used for remedy preparation.

### Medicine preparation, routes of administration, and dosage

Preparations of TMs from plant parts involved boiling (about 27.9%), concoction (about 15.5%), cold maceration, pounding, and decoction. All the TMs were prepared from fresh plant materials for immediate application, as healers believed that fresh materials are more efficacious than stored ones. Water was the most common solvent used, and in some preparations, honey (7.4%), salt (2.9%), and sugar (1.5%) were added to it. The THs claimed that these additives either improve the flavor and taste or reduce the toxicity of the medicine.

**Fig. 3:**
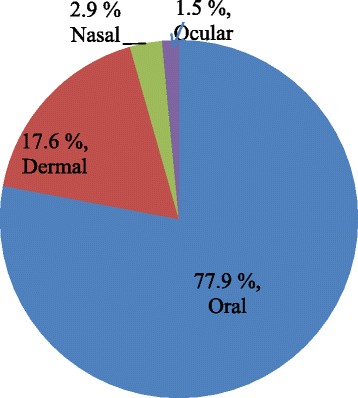
Routes of delivery of the remedy by TM practitioners of the study area

The most common route was oral (77.9%) and this is followed by dermal application (17.6%) (Fig. 3). Oral route involved drinking (64.1%), eating (18.9%), chewing (13.2%), and drenching (3.8%).

The dosage of the remedy given by the THs was determined by inquiring about the age, fitness status, health history, the duration of the illness, presence or absence of pregnancy in women, and other related factors. In case of sensing possible overdosage, the THs recommend a cup of milk, which they believe to minimize possible side effect(s). The measurements of dosage reported include cup, fist, palm, spoon, drops, fingers, and any locally available measuring item. The amount of the MP chewed, swallowed, eaten, or drenched and the duration of remedy application are inconsistent among the THs.

### Traditional MP species used for the treatment of malignancies, jaundice, and helminthiases

Based on the frequently reported diseases, identification and ranking of major MPs used for the treatment of various forms of malignancies/cancers, jaundice (especially in children), and deworming in helminthiases are summarized in Tables 4, 5, and 6. Table 4 lists 14 MPs used to treat malignancies, whereas Table 5 lists 5 MPs claimed to have anti-jaundice activity. Table 6 lists 6 MPs used to deworm in helminthiases, including *Hagenia abyssinica*, the well-known taenicide [34, 35].

**Table 4:** MPs used to treat various forms of malignancies in Megera and adjacent kebeles

Family	Scientific name	Vernacular name (Sidamu-afoo)	Type of cancer treated	Distribution in the flora region	Altitude (meter)	Reference—flora of Ethiopia and Eritrea
Euphorbiaceae	*Ricinus communis* L	Qombo”o	Breast cancer	TU, GD, WU, SU, WG, IL, KF, GG, SD, BA, HA	400-2500	Vol. 2; part II [76]
Fabaceae	*Millettia ferruginea* (Hochst.) Bak.	Hengeddicho	Cancer, in general	WG, SU, HA, BA, IL, KF, SD	1600-2500	Vol. 3 [51]
Flacourtiaceae	*Dovyalis abyssinica* (A. Rich.) Warb.	Shiilo	Lymphatic tumor	TU, GD, GJ, WU, SU, AR, GG, SD, BA, HA	1700-3000	Vol. 2; part 1 [77]
Lamiaceae	*Clerodendrum myricoides* (Hochst.) Vatke	Ma’niisa	Leukemia	TU, GD, WU, SU, AR, WG, IL, KF, GG, SD, HA	700-2600	Vol. 5 [78]
Loganiaceae	*Buddleja polystachya* Fresen.	Bullaancho	Cancer, in general	?AF, TU, GD, GJ, WU, SU, AR, WG, KF, SD, BA, HA	700-3300	Vol. 4; part I [79]
Meliaceae	*Ekebergia capensis* Sparrm.	Goddiicho	Cancer, in general	TU, WU, GD, GJ, WG, SU, AR, IL, KF, SD, BA, HA	1680-3000	Vol. 3 [51]
Melianthaceae	*Bersama abyssinica* Fresen.	Xeweerrakko	Cancer, in general	TU, GD, WU, WG, GJ, SU, IL, KF, AR, HA, BA, SD	1700-2715	Vol. 3 [51]
Myrsinaceae	*Myrsine* melanophloeos (L.) R. Br.	Morocho	Leukemia	TU, GD, GJ, SU, AR, WG, GG, BA	2500-3750*	Vol. 4; part I [79]
Oleaceae	*Olea capensis* L. f.	Seettaame	Cancer, in general	GD, SU, AR, IL, KF, SD, BA	1350-3200	Vol. 4; part I [79]
Ranunculaceae	*Clematis simensis* Fresen.	Fiide	Cancer, in general	GD, TU, WU, GJ, SU, AR, WG, KF, GG, SD, BA, HA,	1500-3350	Vol. 2; part I [77]
Rubiaceae	*Pentas lanceolata* Forssk.	Baalaamu dhagga	Cancer, in general	TU, GD, IL, KF GG, SD, BA, HA	700-2300	Vol. 4; part I [79]
Rutaceae	*Zanthoxylum chalybeum* Engl.	Gadda	Cancer, in general	GG, BA, HA	900-1550*	Vol. 3 [51]
Sapotaceae	*Sideroxylon oxyacanthum* Baill.	Bunguude	Cancer, in general	TU GD, SU, AR, BA, HA	1250-2800*	Vol. 4; part I [79]
Zingiberaceae	*Zingiber officinale* Roscoe	Jaanjiweelo	Cancer, in general	IL, KF, SD	NA	Vol. 6 [80]

*Abbreviations*: *TU* Tigray region above 1000 m contour, *AF* Afar region below 1000 m contour to Eritrean border in the east and Harerge border in the south, *WU* Welo region above 1000 m contour, *GD* Gondar region, *WG* Welega region, *KF* Kefa region, *AR* Arsi region, *BA* Bale region, *GJ* Gojam region, *IL* Ilubabor region, *GG* Gamo Gofa region, *SD* Sidamo region, *HA* Harerge region, *NA* not available

*Not found in the range reported in the SD flora region

**Table 5:** MPs used to treat jaundice in Megera and adjacent kebeles

Family	Scientific name	Vernacular name (Sidamu-afoo)	Distribution in the flora region	Altitude (meter)	Reference—flora of Ethiopia and Eritrea
Cucurbitaceae	*Peponium vogelii* (Hook.f.) Engl.	Surupha	SU IL, KF, SD, BA	1500-2100	Vol. 2; part II [76]
Cuppressaceae	*Juniperus procera* Hochst ex. Engl.	Hoomme	TU, GD, GJ, WU, SU, AR, SD, HA	1100-3500	Vol. 1 [81]
Menispermaceae	*Stephania abyssinica* Dillon and A.Rich.	Kalaala	TU, GD, GJ, WU, SU, AR, WG, KF, IL, GG, SD, HA	1450-3400	Vol. 2; part I [77]
Podocarpaceae	*Podocarpus falcatus* (Thunb.) Mirb.	Dagucho	TU, GD, GJ, WU, SU, AR, IL, KF, GG, WG, SD, BA, HA	1350-2900	Vol. 1 [81]
Polygonaceae	*Rumex abyssinicus* Jacq.	Shiishoone	TU, GD, GJ, SU, AR, WG, KF, IL, GG, SD, BA, HA	1200-3300	Vol. 2; part I [77]

*Abbreviations*: *TU* Tigray region above 1000 m contour, *AF* Afar region below 1000 m contour to Eritrean border in the east and Harerge border in the south, *WU* Welo region above 1000 m contour, *GD* Gondar region, *WG* Welega region, *KF* Kefa region, *AR* Arsi region, *BA* Bale region, *GJ* Gojam region, *IL* Ilubabor region, *GG* Gamo Gofa region, *SD* Sidamo region, *HA* Harerge region

**Table 6:** MPs used to deworm in helminthiases in Megera and adjacent kebeles

Family	Scientific name	Vernacular name (Sidamu-afoo)	Distribution in the flora region	Altitude (meter)	Reference—flora of Ethiopia and Eritrea
Acanthaceae	*Hypoestes forskaolii* (Vahl) R. Br.	Ciikkicho	AF, EW, TU, GD, GJ, WU, SU, AR, WG, IL, KF, GG, SD, BA, HA,	400-2900	Vol. 5 [78]
Asteraceae	*Vernonia amygdalina* Del.	Hechcho	TU, ?WU, GD, GJ, SU, WG, IL, KF, GG, SD, BA, HA	650-3000	Vol. 4; part II [82]
Lamiaceae	*Plectranthus garckeanus* (Vatke) J.K. Morton	Toontoona	GD, GJ, SU, AR, KF, SD, BA	1750-2700	Vol. 5 [78]
Phytolaccaceae	*Phytolacca dodecandra* L’Herit.	Haraanjicha	TU, BA, GG, GD, WU, GJ, WG, SU, IL, KF, AR, SD, HA	1500-3000	Vol. 2; part I [77]
Polygonaceae	*Rumex nervosus* Vahl	Taare	TU, GD, GJ, WU, SU, AR, GG, SD, HA	400-3300	Vol. 2; part I [77]
Rosaceae	*Hagenia abyssinica* (Bruce) J.F. Gmel (hagenia)	So”ichote dhagga (qaanqo)	TU, GD, WU, GJ, WG, SU, AR, BA, HA, KF, SD	2450-3250	Vol. 3 [51]

*Abbreviations*: *TU* Tigray region above 1000 m contour, *AF* Afar region below 1000 m contour to Eritrean border in the east and Harerge border in the south, *WU* Welo region above 1000 m contour, *GD* Gondar region, *WG* Welega region, *KF* Kefa region, *AR* Arsi region, *BA* Bale region, *GJ* Gojam region, *IL* Ilubabor region, *GG* Gamo Gofa region, *SD* Sidamo region, *HA* Harerge region

The analysis of preferences of THs among five frequently cited traditional MPs used to treat various malignancies/cancers showed that *Sideroxylon oxyacanthum* was the most preferred species (26), followed by *Zanthoxylum chalybeum* (24) (Table 7). *Podocarpus falcatus* was the most preferred traditional MP to treat jaundice followed by *Rumex abyssinicus* (Table 8)*.* To deworm in helminthiases, *Hagenia abyssinica* was the most widely used plant by herbalists of the study area followed by *Rumex nervosus* (Table 9)*.*

**Table 7:** Preference ranking of MPs used to treat malignancies in humans

Informants	MPs				
	*Zanthoxylum chalybeum* (Gadda)	*Olea capensis* (Seettaame)	*Bersama abyssinica* (Xeweerrakko)	*Sideroxylon oxyacanthum* (Bunguude)	*Dovyalis abyssinica* (Shiilo)
1	4	1	2	5	3
2	4	3	1	5	2
3	5	2	1	4	3
4	2	1	5	3	4
5	4	2	3	5	1
6	5	1	2	4	3
Total	24	10	14	26	16
Rank	2nd	5th	4th	1st	3rd

**Table 8:** Preference ranking of MPs used to treat jaundice in human

Informants	MPs				
	*Rumex abyssinicus* (Shiishoone)	*Podocarpus falcatus* (Dagucho)	*Stephania abyssinica* (Kalaala)	*Peponium vogelii* (Surupha)	*Juniperus procera* (Hoomme)
1	4	5	2	1	3
2	4	5	1	2	3
3	3	4	1	2	5
4	5	3	4	1	2
5	4	5	1	2	3
6	3	5	4	1	2
Total	23	27	13	9	18
Rank	2nd	1st	4th	5th	3rd

**Table 9:** Preference ranking of MPs used to deworm in helminths in humans

Informants	MPs				
	*Rumex nervosus* (Taare)	*Hagenia abyssinica* (So”ichote dhagga)	*Plectranthus garckeanus* (Toontoona)	*Hypoestes forskaolii* (Ciikkicho)	*Rubus apetalus* (Go’ra)
1	4	5	3	1	2
2	3	5	4	1	2
3	3	4	5	2	1
4	4	5	2	3	1
5	3	4	2	1	5
6	4	5	2	1	3
Total	21	28	18	9	14
Rank	2nd	1st	3rd	5th	4th

### Degree of THs’ agreement on herbal medicines

MP selection by the THs depends heavily on their prior acquisition of knowledge from their parents or through lateral communication with other herbalists in the community on the basis of mutual benefit. Furthermore, healers held that historical anecdotes were taken into account in choosing the most effective MPs and to avoid the plants that are reported to have serious side effects. From the reports, it was seen that some plants (e.g., *Ocimum lamiifolium* and *Salvia nilotica*) were used by all of the THs who participated in the study while other plants were known only by a small fraction of the herbalists. The calculation of the ICF values for the highly prevalent ailments including various forms of malignancies/cancer, jaundice, helminthiases, *‘miche’*, various forms of wound, sudden illnesses (*‘lammootta*’ or *‘dingetegna*’), and malaria was performed. TMs used to treat *‘miche’* had the highest ICF of 0.97 and the lowest 0.86 for malignancies/cancer, according to the THs (Table 10).

**Table 10:** Results of informants consensus factor (ICF) for most prevalent human illnesses in Megera and adjacent kebeles

Ailment treated	List of plants used [Species (number of reports)]s	Total number of species	Total number of reports	ICF
*‘Miche*’	*Ocimum lamiifolium* (20), *Salvia nilotica* (20)	2	40	0.97
Jaundice (liver disease)	*Juniperus procera* (6), *Peponium vogelii* (5), *Podocarpus falcatus* (13), *Rumex abyssinicus* (17), *Stephania abyssinica* (3)	5	44	0.91
Wounds	*Pentas lanceolata* (6), *Plectranthus punctatus* (15), *Solanum aculeatissimum* (8), *Solanum dasyphyllum* (7), *Vernonia auriculifera* (12)	5	48	0.91
Malaria	*Croton macrostachyus* (14), *Peponium vogelii* (5), *Solanum nigrum* (5), *Vernonia amygdalina* (11)	4	35	0.91
Sudden illnesses (*‘lammootta*’ or *‘dingetegna*’)	*Aloe* sp.(12), *Rumex nervosus* (3), *Prunus persica* (5)	3	20	0.89
Helminthiases	*Hagenia abyssinica* (16), *Hypoestes forskaolii* (3), *Phytolacca dodecandra* (5), *Plectranthus garckeanus* (5), *Rumex nervosus* (4), *Vernonia amygdalina* (10)	6	43	0.88
Malignancies/cancer	*Bersama abyssinica* (9), *Buddleja polystachya* (3), *Clematis simensis* (7), *Clerodendrum myricoides* (4), *Dovyalis abyssinica* (13), *Ekebergia capensis* (5), *Millettia ferruginea* (6), *Olea capensis* (11), *Pentas lanceolata* (2), *Myrsine melanophloeos* (4), *Ricinus communis* (2), *Sideroxylon oxyacanthum* (15), *Zanthoxylum chalybeum* (10), *Zingiber officinale* (3)	14	94	0.86

### MPs with high comparative advantages against malignancies

From among the most frequent diseases where patients in the study area usually showed more preferences to THs over modern healthcare, according to THs, was malignancies/cancer of various types. Hence, pair-wise exercise was undertaken with five THs who used their knowledge of treating patients with malignancies and compared five MPs, and the results are shown in Table 11. It can be seen that *Sideroxylon oxyacanthum* was picked as the best TM to treat malignancies followed by *Zanthoxylum chalybeum*. *Bersama abyssinica* was the least popularly picked MP among the topmost species. In both preference ranking and pair-wise comparison matrices, two plant species were topping the lists, *Sideroxylon oxyacanthum* and *Zanthoxylum chalybeum*, and these species were also among those that had high informant consensus.

**Table 11:** Pairwise comparison of MPs used to treat malignancies

Informants (Is)	MPs				
	*Zanthoxylum chalybeum* (Gadda)	*Dovyalis abyssinica* (Shiilo)	*Olea capensis* (Seettaame)	*Sideroxylon oxyacanthum* (Bunguude)	*Bersama abyssinica* (Xeweerrakko)
I_1_	3	2	1	4	0
I_2_	2	3	3	4	2
I_3_	4	2	1	3	1
I_4_	1	0	2	4	2
I_5_	3	1	2	2	1
Total	13	8	9	17	6
Rank	2nd	4th	3rd	1st	5th

### Fidelity level of anticancer herbal medicines

The proportion of informants claiming the widespread use of *Sideroxylon oxyacanthum*, *Zanthoxylum chalybeum*, and *Dovyalis abyssinica* against malignancies was 70% for *Sideroxylon oxyacanthum*, 55% for *Zanthoxylum chalybeum*, and 45% for *Dovyalis abyssinica*. The highest informant consensus for *Sideroxylon oxyacanthum* indicates that it is perceived as the best MP to treat malignancies.

### Plants with multiple medicinal uses

Most (59.2%) MPs reported from the study area were used to treat single illness. However, about 40.8% of the MPs were reported to have medicinal roles for two or more illnesses (Table 12) and with 58.3% of the remedies for different illnesses prepared from the same part of the MP. However, only 37.5% of the remedies with multiple medicinal uses were reported to have the same method of preparation, implying that the same part of the plant can be used for various ailments when prepared in a different way.

**Table 12:** MPs used to treat two or more human ailments

Family	Scientific name	Vernacular name (Sidamu-afoo)	PU
Acanthaceae	*Hypoestes forskaolii* (Vahl) R. Br.	Ciikkicho	LRt
Alliaceae	*Allium sativum* L.	Waajjo tuma	Bu
Anacardiaceae	*Mangifera indica* L.	Mango	Lt
Apocynaceae	*Acokanthera schimperi* (A. DC.) Schweinf.	Qaraaro	Fr
Asparagaceae	*Asparagus africanus* Lam.	Buticho	Rt
Asteraceae	*Vernonia amygdalina* Del.	Hechcho	Lf
Caricaceae	*Carica papaya* L.	Paappaye	LFr
Clusiaceae	*Garcinia buchananii* Baker	Soloolsa	Ba
Cucurbitaceae	*Cyclantheropsis parviflora* (Cogn.) Harms	Basu baaqula	FrS
	*Peponium vogelii* (Hook. f.) Engl.	Surupha	Fr
Cuppressaceae	*Juniperus procera* Hochst ex. Engl.	Hoomme	LSh
Dioscoreaceae	*Dioscorea alata* L.	Bohe	LTb
Icacinaceae	*Apodytes dimidiata* E. Mey. ex Arn.	Doongicho	Ba
Lamiaceae	*Plectranthus garckeanus* (Vatke) J.K. Morton	Toontoona	Lf
Meliaceae	*Ekebergia capensis* Sparrm.	Goddiicho	Fr
Musaceae	*Ensete ventricosum* (Welw*.*) Cheesman	Weese	STb
	*Musa paradisiaca* L.	Muuze	Lt
Myrtaceae	*Syzygium guineense* (Wild.) DC.	Duuwancho	BFr
Phytolaccaceae	*Phytolacca dodecandra* L’Herit.	Haraanjicha	Lf
Podocarpaceae	*Podocarpus falcatus* (Thunb.) Mirb.	Dagucho	SLt
Polygonaceae	*Rumex nervosus* Vahl	Taare	Rt
Ranunculaceae	*Clematis simensis* Fresen.	Fiide	LSt
Rhamnaceae	*Gouania longispicata* Engl.	Daanikuukke	Lf
Rosaceae	*Rubus apetalus* Poir.	Go’ra	Rt
Rubiaceae	*Pentas lanceolata* Forssk.	Baalaamu dhagga	Rt
Rutaceae	*Ruta chalepensis* L.	Xenaddaame	Sh
	*Zanthoxylum chalybeum* Engl.	Gadda	LSh
Solanaceae	*Solanum* aculeatissimum Jacq.	Haanja borbodho	LSh
Zingiberaceae	*Zingiber officinale* Rosc.	Jaanjiweelo	RRz

*PU* part used (*Br* bark, *Bu* bulb, *BFr* bark and fruit, *Fr* fruit, *FrS* fruit and seed, *Lt* latex, *Lf* leaf, *LFr* leaf and fruit, *LSt* leaf and stem, *LRt* leaf and root, *LSh* leaf and shoot, *LTb* leaf and tuber, *Rt* root, *RRz* root and rhizoid, *Sh* shoot, *SLt* shoot and latex, *STb* stem and tuber)

### Market values of the MPs

The majority (77.5%) of MPs reported from the study area were limited to usage only when processed and given by THs. Therefore, they were not sold in the open markets for their medicinal values. However, it was documented during the market survey that some of the MPs were known for their other use values and were sold in the local markets. These plants included *Allium sativum*, *Zingiber officinale*, and *Ruta chalepensis*, marketed as spices; *Citrus aurantifolia*, *Carica papaya*, *Ensete ventricosum* (the staple food in the area), *Garcinia buchananii*, *Mangifera indica*, *Musa x paradisiaca*, *Passiflora edulis*, *Prunus persica*, and *Solanum nigrum*, sold as foodstuff. Other marketable plants included *Olea europaea* (a fumigant for utensils to keep dairy products and for pleasant smell of traditional houses), *Rhamnus prinoides* (as an additive to fermented beverages), *Coffea arabica* (for drinking as hot beverage), and *Ricinus communis* for smearing the *‘injera*’(traditional thin, spongy bread made in Ethiopia) baking plates*.*

### Secrecy of TMs among the practitioners

TM practitioners of Megera and adjacent kebeles were found to keep their knowledge of traditional herbal medicines away from the community to retain its secrecy. Especially, the elderly practitioners collect and process the remedy in strict secrecy. They asserted that they do this in order to sustain the income they earn by giving the remedy for the customers who consult them for their health problems. Some also mentioned a belief that if the plant is widely known by the community, the potency of the plant will be lost. Some also associate the secrecy of the traditional medicinal knowledge with rituals of gods, claiming that the gods do not allow disclosing the MPs as long as they live healthily on the face of the earth [Personal communication, Kalaa Ilaala Wobbisa].

## Discussion

TM is an important and often underestimated part of healthcare, and it is practiced in almost every country in the world, and demand for its services is currently increasing in the form of alternative medicine. It is the main source of healthcare, and sometimes the only source of care, due to its closeness to the ordinary rural communities and its accessibility and affordability in view of the rising healthcare costs [36]. Ethiopians, by and large, depend on TM for their primary healthcare due to limited access to functional modern healthcare facilities, affordability, cultural acceptability of healers, perceived efficacy against certain types of diseases, and the belief that TM has low side effects [15, 37–39]. As a result of improved access to modern medicines and environmental degradation, both traditional knowledge and the plants that have been in use as medicines, for millennia, are highly threatened. However, in spite of this environmental degradation by anthropogenic activities and periodic droughts, there are still many MP species in different parts of Ethiopia [12, 38, 40–42]. And the findings of the present study in a highly limited area in Sidama Zone, where 71 species of medicinally useful plants are in use for the treatment of various human ailments, are an indication of the potential for the conservation of MPs in the country.

Shrubs make a larger proportion of the MPs used in the study area, and this finding agrees with previous reports [13, 43–46]. This could be attributed to the common pattern of growth forms in the study area [47]. However, reports elsewhere showed that herbs were the frequently used MPs, which have a higher relative abundance of herbs as compared to other life forms [48–50]. This could further relate to the abundance of areas with bushy vegetation in the first case and less woody species in the second.

The present findings revealed that the families Lamiaceae (six species), Asteraceae, Euphorbiaceae, and Rubiaceae (four species each) encompass more numbers of MP species. These families are among the MP families topping the list in other reports from different parts of the country, and their higher proportion in the flora of Ethiopia substantiates this report, implying documentation of wide distribution and higher number of species of these families throughout the country [13, 38, 51, 52].

In the present study, it was determined that 19.7% of the MPs were used to treat various forms of malignancies. Reports from other parts of the country also substantiate the present finding [13, 20]. Plants have a reputable contribution to the discovery of anticancer bioactive compounds [53]. Polyphenols, brassinosteroids, and taxols, among others, have experimentally proven anticancer activities [53–55].

It was also found that the most widely used part of the MPs was the leaf, and this finding agrees with previous reports [19, 42, 49, 52, 56–62]. Naturally, the plants are surrounded by a series of potential threats, and evolutionarily, they have developed strategies of defense against the attacks of various pathogens (e.g., bacteria, viruses, fungi, nematodes, mites, insects), herbivores, and various kinds of abiotic stresses [63]. Therefore, plants produce ranges of organic compounds known as secondary metabolites, which a priori is not directly involved in their growth and development. Arguably, the leaves are exposed more to the enemies, and these chemicals, therefore, play a defensive role [64, 65]. Thus, an approach of using leaves as a major part of a plant for a remedy preparation could be taken as an indicator of scientific relevance. It also is noteworthy that zoopharmacognostic approach (observation of animal self-medication behavior) where animals consume mainly leaves of certain plants had led historically to the discovery of some aliphatic drugs, and this further establishes the preferential use of leaves as a source of TMs [66, 67]. Furthermore, harvesting aerial parts, such as leaves, poses less threat to the MPs when compared to exploiting the roots and barks, as also argued in other reports [42, 58, 68].

In the study area, all the TMs are said to be used preferably in their fresh forms, and this may compromise the sustainability of the MPs in the long run as it would be exploited extensively every time the remedy will have to be prepared. Other researchers [58, 69] have also expressed similar concerns.

Since the majority (64.7%) of the MPs collected from the study area were found in the wild habitats, the high risk of MP destruction by anthropogenic activities, on top of ensuing climate change, is a matter of serious concern. The anthropogenic activities driven by population growth are real and put pressure on natural resources. These include agricultural expansion, tree cutting for urbanization, house construction, firewood, fodder, drought, overgrazing, and charcoal making which serve as major threats to the MPs [46, 59, 70].

It was found that THs of the study area keep the MPs in strict secrecy and transfer their knowledge to their trustworthy elder sons only at an advanced age. This is a matter of concern because in the process, there might be misrepresentation, distortion, incompleteness, or omission of the original information as this is done orally [40, 52]. Though keeping the MPs away from the home gardens is believed to ensure the secrecy by the practitioners, it renders the MPs to grazing and unsustainable use [46]. Conversely, one report argues that keeping the MPs in strict secrecy contributed to sustaining the MPs [45], and it has an added advantage of safety against improper self-medication, which could be dangerous in the case of some plants.

The finding that 15.5% of the MP preparations were made by mixing two or more plants as a single remedy, could be explained by the assumption that a cocktail of plant material would be more effective in addition to maintaining the secrecy of the MPs. Furthermore, Megersa and co-workers [58] had reported that using such concoction is believed to increase the strength and efficacy of the remedy. Others have also argued that mixing two or more remedies helps to avoid or minimize possible side effect(s) [13].

The reports of the THs have also indicated the toxicity of some MPs, such as *Hagenia abyssinica*, a widely used anti-helminthic MP, which also was corroborated by other reports [35]. Reports from laboratory animal tests have shown that many reputable plants are medicinally used for centuries to have potential toxicity on blood parameters and histopathology of internal organs [71]. Therefore, it is recommendable that in vivo and in vitro toxicity investigations on the frequently used TMs must be conducted to avoid possible toxic effects of the MP reported in the present study.

Furthermore, one of the challenges identified by the present study was the inconsistency of the dosage from one preparation to the other even for the same kind of illness. However, in some cases, these variations are in relation to age, physical status, the course of illness, presence or absence of pregnancy, and other concomitant illness as also reported by earlier workers [42, 46, 50, 58]. Thus, the lack of precise dosage is one of the drawbacks of traditional medicine practices, as also emphasized by others [12, 72].

The community of the study area relies on mixed agricultural practices involving both crop farming and livestock rearing. Due to a shortage of farmland, farm expansion into the woodlands is affecting the vegetation cover and hence the MPs in the area. Furthermore, cattle, sheep, and goats compose main livestock reared in the area, and some of the MPs are collected as forage for the livestock, as also reported elsewhere, and goats are known to forage on open fields on various MPs [58]. Thus, the reality of existential threats to the sustainability of valuable MPs of the area is a serious one.

One of the greatest challenges in ethnomedicinal researches is the issue of access and benefit sharing (ABS). There have been attempts to implement ABS system of the Convention on Biological Diversity (CBD) in Ethiopia, taking into account the international obligations, including Nagoya Protocol, and other national considerations [73, 74]. The Nagoya Protocol, which came into effect on 12 October 2014, is an international legal tool that offers protection for traditional knowledge of medicines with the associated rights of indigenous communities [75]. Even though the country has acceded to the Nagoya Protocol and had developed a code of conduct to enforce the ABS provisions, there are several issues which demand thorough action [73]. These encompass, problems related to human resources and institutional capacity building, centralization system, lack of effective enforcement and follow up mechanisms, vagueness of the code of conduct, and lack of effective scheme for community participation in ABS [73, 74]. It would therefore be very appropriate to recommend enforcement of the Nagoya Protocol with all its international recommendations so that the TM knowledge and associated rights of the indigenous communities are fully protected. If this is addressed adequately, it will facilitate the proper documentation of valuable knowledge held by the TM practitioners and contribute to salvaging the loss of traditional herbal medicines.

## Conclusion

The present study compiled the knowledge held by TM practitioners, in addition to assessing the ethnobotanical knowledge available in the public domain. Megera and adjacent kebeles have diverse MP species used to treat various human ailments. The present study has shown that the THs of the area have a rich knowledge of TM and that traditional herbal medicine is an integral part of the healthcare system. Due to very scarce modern healthcare facilities around Megera and the economic insufficiency of the community to meet the prices of available modern healthcare services, THs are the most reliable providers of healthcare services to the community.

The majority (64.7%) of the MPs are found in wild habitats where anthropogenic activities are advancing and hence are vulnerable for destruction. Therefore, sustainable integrated natural resource management system has to be put in place as soon as possible. Indigenous stewardship of the natural resource, including the TM plants, has to be documented and augmented with government and non-government environmental rehabilitation programs to conserve the vegetation of the area. Other plant use categories including foods and drinks, firewood, charcoal making, construction and tools, commercial, bee forage, fodder, shade, cultural rituals, ornamental, etc. must also be documented in order to enhance the conservation of these vital natural resources.

We recommend an in-depth experimental investigation of the frequently cited MPs believed by the THs to have high comparative advantages over others in treating various malignancies, jaundice, and helminthiases. This would possibly contribute to the current global drug development endeavors.

## Abbreviations

ABS: Access and benefit sharing
FL: Fidelity level
ICF: Informant consensus factor
MP/MPs: Medicinal plant(s)
TH/THs: Traditional healer(s)
TM/TMs: Traditional medicine(s)
UV: Use value

## Footnotes

1 Epiphytes are collected by the THs, boiled, and filtered, and the filtrate is given orally to treat emaciated malnourished infants.

2 The term “malignancy” is used to define conditions/diseases in which neoplastic cells divide without control and can invade nearby tissues. It constitutes an enormous burden globally, and the world is facing stern challenge of drug resistance that leads to treatment failure and disease recurrence.

## References

[1] Kassu A, Urga K, Assefa A, Guta M (2004). Ethnobotanical survey and the medicinal plants of some areas in south and central Ethiopia. Traditional medicine in Ethiopia, Proceedings of a National Workshop held in Addis Ababa, Ethiopia, on June 30–2 July.

[2] IBC (2009). Convention on Biological Diversity (CBD) Ethiopia’s 4^th^ Country Report.

[3] http://www.ethiomet.gov.et. Hydro Meteorological Bulletin for May, 2016. http://www.ethiometgovet/bulletins/view_pdf/418/1-31__dec_2015pdf. Accessed 14 Mar 2017.

[4] Awas T, Gashaw M, Tesfaye G, Tihune A (2003). Ecosystems of Ethiopia. National Biodiversity Strategy and Action Plan (NBSAP) project.

[5] Awas T (2007). Plant diversity in western Ethiopia: ecology, ethnobotany and conservation. Doctoral dissertation.

[6] Kelbessa E, Demissew S (2014). Diversity of vascular plant taxa of the flora of Ethiopia and Eritrea. Eth J Biol Sci.

[7] Gebre-Egziabher TB, JMM E, Hawkes JG, Worede M (1991). Diversity of Ethiopian flora. Plant genetic resources of Ethiopia.

[8] Tena R (2014). Endemic medicinal plants of Ethiopia: review of the literature. PhD seminar.

[9] Bekele E (2007). Study on actual situation of medicinal plants in Ethiopia.

[10] Desta B. Ethiopian traditional herbal drugs: potentiality and appropriate utilization. In: 8^th^ International Conference of Ethiopian Studies. Addis Ababa; 1984. p. 763–6.

[11] Jansen PCM (1981). Spices, condiments and medicinal plants in Ethiopia, their taxonomy and agricultural significance.

[12] Dawit A (1986). Traditional medicine in Ethiopia: the attempts being made to promote it for effective and better utilization. SINET: Eth J Sci.

[13] Kefalew A, Asfaw Z, Kelbessa E (2015). Ethnobotany of medicinal plants in Ada’a District, East Shewa Zone of Oromia regional state, Ethiopia. J Ethnobiol Ethnomed.

[14] Salaverry O (2013). Back to the roots: traditional medicine for cancer control in Latin America and the Caribbean. Lancet Oncol..

[15] Birhan W, Giday M, Teklehaymanot T (2011). The contribution of traditional healers’ clinics to public health care system in Addis Ababa, Ethiopia: a cross-sectional study. J Ethnobiol Ethnomed.

[16] Woldeamanuel YW, Girma B, Teklu AM (2013). Cancer in Ethiopia. Lancet Oncol.

[17] Asnake S, Teklehaymanot T, Hymete A, Erko B, Giday M. Survey of medicinal plants used to treat malaria by Sidama people of Boricha District, Sidama Zone, south region of Ethiopia. Evid Based Complement Alternat Med. 2016; 10.1155/2016/9690164:9690164. 10.1155/2016/9690164 PMC4775815 26989429

[18] Kewessa G, Abebe T, Demessie A. Indigenous knowledge on the use and management of medicinal trees and shrubs in Dale District, Sidama Zone, southern Ethiopia. Ethnobot Res Appl. 2015;14:171–182.

[19] Regassa R (2013). Assessment of indigenous knowledge of medicinal plant practice and mode of service delivery in Hawassa city, southern Ethiopia. J Med Plants Res.

[20] Abate G. Etse Debdabe – Ethiopian traditional medicine (Amharic version). Edited by Demissew S: Addis Ababa University, Addis Ababa, Ethiopia; 1989.

[21] CSA (2013). Population projection of Ethiopia for all regions at Wereda level from 2014–2017.

[22] SZFaED Office. Finance and economic development office (FaED) of Sidama Zone. Hawassa; 2015.

[23] Dale District Health Department. A summary of top ten human infections reported from health centers of the woreda (annual report summary). Yirgalem; 2015.

[24] McClatchey W, Gollin LX (2005). An ethnobotany research training workshop in Madagascar. Ethnobot Res Appl.

[25] Hedberg I (1993). Botanical methods in ethnopharmacology and the need for conservation of medicinal plants. J Ethnopharmacol.

[26] Martin GJ (1995). Ethnobotany: a methods manual.

[27] Cotton CM (1996). Ethnobotany: principles and applications.

[28] Heinrich M, Ankli A, Frei B, Weimann C, Sticher O (1998). Medicinal plants in Mexico: healers’ consensus and cultural importance. Soc Sci Med.

[29] Amiguet VT, Arnason JT, Maquin P, Cal V, Vindas PS, Poveda L (2005). A consensus ethnobotany of the Q’eqchi’Maya of southern Belize. Econ Bot.

[30] Gazzaneo LRS, De Lucena RFP, de Albuquerque UP (2005). Knowledge and use of medicinal plants by local specialists in an region of Atlantic Forest in the state of Pernambuco (northeastern Brazil). J Ethnobiol Ethnomed.

[31] Alexiades MN (1996). Collecting ethnobotanical data: an introduction to basic concepts and techniques. Adv Econ Bot.

[32] Prance GT, Baleé W, Boom B, Carneiro RL (1987). Quantitative ethnobotany and the case for conservation in ammonia. Conserv Biol.

[33] Andrade-Cetto A, Heinrich M. From the field into the lab: useful approaches to selecting species based on local knowledge. Front Pharmacol. 2011;2:20. 10.3389/fphar.2011.00020 PMC3108584 21954385

[34] Pankhurst R (1969). The traditional taenicides of Ethiopia. J Hist Med Allied Sci.

[35] Assefa B, Glatzel G, Buchmann C (2010). Ethnomedicinal uses of Hagenia abyssinica (Bruce) JF Gmel. among rural communities of Ethiopia. J Ethnobiol Ethnomed.

[36] WHO (2013). World Health Organization: traditional medicine strategy 2014–2023.

[37] Kassaye KD, Amberbir A, Getachew B, Mussema Y (2006). A historical overview of traditional medicine practices and policy in Ethiopia. Ethiop J Health Dev.

[38] Belayneh A, Bussa NF (2014). Ethnomedicinal plants used to treat human ailments in the prehistoric place of Harla and Dengego valleys, eastern Ethiopia. J Ethnobiol Ethnomed.

[39] Tolossa K, Debela E, Athanasiadou S, Tolera A, Ganga G, Houdijk JG (2013). Ethno-medicinal study of plants used for treatment of human and livestock ailments by traditional healers in South Omo, southern Ethiopia. J Ethnobiol Ethnomed.

[40] Debela A, Abebe D, Urga K (1999). An over view of traditional medicine in Ethiopia: perspective and developmental efforts. Ethiop Pharmaceut Ass.

[41] Pankhurst R. An introduction to the medical history of Ethiopia. Trenton: The Red Sea Press, Inc; 1990.

[42] Teklay A, Abera B, Giday M (2013). An ethnobotanical study of medicinal plants used in Kilte Awulaelo District, Tigray region of Ethiopia. J Ethnobiol Ethnomed.

[43] Yineger H, Yewhalaw D (2007). Traditional medicinal plant knowledge and use by local healers in Sekoru District, Jimma Zone, southwestern Ethiopia. J Ethnobiol Ethnomed.

[44] Lulekal E, Kelbessa E, Bekele T, Yineger H (2008). An ethnobotanical study of medicinal plants in Mana Angetu district, southeastern Ethiopia. J Ethnobiol Ethnomed.

[45] Mesfin F, Demissew S, Teklehaymanot T (2009). An ethnobotanical study of medicinal plants in Wonago Woreda, SNNPR, Ethiopia. J Ethnobiol Ethnomed.

[46] Bekele G, Reddy PR (2015). Ethnobotanical study of medicinal plants used to treat human ailments by Guji Oromo tribes in Abaya District, Borana, Oromia. Ethiopia Uni J Plant Sci.

[47] Friis I, Demissew S, Breugel PV (2010). Atlas of the potential vegetation of Ethiopia. Det Kongelige Danske Videnskabernes Selskab.

[48] Teklehaymanot T, Giday M (2007). Ethnobotanical study of medicinal plants used by people in Zegie Peninsula, northwestern Ethiopia. J Ethnobiol Ethnomed.

[49] Bekalo TH, Woodmatas SD, Woldemariam ZA (2009). An ethnobotanical study of medicinal plants used by local people in the lowlands of konta special woreda, southern nations, nationalities and peoples regional state, Ethiopia. J Ethnobiol Ethnomed.

[50] Birhane E, Aynekulu E, Mekuria W, Endale D (2011). Management, use and ecology of medicinal plants in the degraded dry lands of Tigray, northern Ethiopia. J Med Plants Res.

[51] Hedberg I, Edwards S (1989). Flora of Ethiopia, volume 3: Pittosporaceae to Araliaceae: the National Herbarium.

[52] Chekole G, Asfaw Z, Kelbessa E (2015). Ethnobotanical study of medicinal plants in the environs of Tara-gedam and Amba remnant forests of Libo Kemkem District, northwest Ethiopia. J Ethnobiol Ethnomed.

[53] Xie S, Zhou J. Harnessing plant biodiversity for the discovery of novel anticancer drugs targeting microtubules. Front Plant Sci. 2017;8 10.3389/fpls.2017.00720 PMC5415602 28523014

[54] Tewari T, Singh R, Pant V, Kumar A, Chaturvedi P. Plant-Derived Compounds with Anticancer Properties: From Folklore to Practice. In: Malik S., editor. Biotechnology and Production of Anti-Cancer Compounds. Cham: Springer; 2017. p. 99–119.

[55] Tafrihi M, Nakhaei SR. E-cadherin/β-catenin complex: a target for anticancer and antimetastasis plants/plant-derived compounds. Nutr Cancer. 2017; 10.1080/01635581.2017.1320415. 10.1080/01635581.2017.1320415 28524727

[56] Suleman S, Alemu T (2012). A survey on utilization of ethnomedicinal plants in Nekemte town, east Wellega (Oromia), Ethiopia. J Herbs Spices Med Plants.

[57] Agize M, Demissew S, Asfaw Z (2013). Ethnobotany of medicinal plants in Loma and Gena bosa districts (woredas) of dawro zone, southern Ethiopia. Topcls J Herb Med.

[58] Megersa M, Asfaw Z, Kelbessa E, Beyene A, Woldeab B (2013). An ethnobotanical study of medicinal plants in Wayu Tuka district, east Welega zone of oromia regional state, West Ethiopia. J Ethnobiol Ethnomed.

[59] Araya S, Abera B, Giday M (2015). Study of plants traditionally used in public and animal health management in Seharti Samre District, southern Tigray, Ethiopia. J Ethnobiol Ethnomed.

[60] Seid MA, Tsegay BA (2011). Ethnobotanical survey of traditional medicinal plants in Tehuledere district, South Wollo, Ethiopia. J Med Plants Res.

[61] Enyew A, Asfaw Z, Kelbessa E, Nagappan R (2014). Ethnobotanical study of traditional medicinal plants in and around Fiche District, central Ethiopia. Curr Res J Biol Sci.

[62] Zerabruk S, Yirga G (2012). Traditional knowledge of medicinal plants in Gindeberet district, western Ethiopia. S Afr J Bot.

[63] Piasecka A, Jedrzejczak-Rey N, Bednarek P (2015). Secondary metabolites in plant innate immunity: conserved function of divergent chemicals. New Phytol.

[64] Olivoto T, Nardino M, Carvalho IR, Follmann DN, Szareski V, Jardel I, Ferrari M (2017). Plant secondary metabolites and its dynamical systems of induction in response to environmental factors: a review. Afr J Agric Res.

[65] Bartwal A, Mall R, Lohani P, Guru S, Arora S (2013). Role of secondary metabolites and brassinosteroids in plant defense against environmental stresses. J Plant Growth Regul.

[66] Shurkin J (2014). News feature: animals that self-medicate. Proc Natl Acad Sci.

[67] Katiyar C, Gupta A, Kanjilal S, Katiyar S (2012). Drug discovery from plant sources: an integrated approach. Ayu.

[68] Ragunathan M, Abay SM (2009). Ethnomedicinal survey of folk drugs used in Bahirdar Zuria District, northwestern Ethiopia. Indian J Trad Knowl.

[69] Maryo M, Nemomissa S, Bekele T (2015). An ethnobotanical study of medicinal plants of the Kembatta ethnic group in enset-based agricultural landscape of Kembatta Tembaro (KT) Zone, southern Ethiopia. Asian J Plant Sci Res.

[70] Eshete MA, Kelbessa E, Dalle G (2016). Ethnobotanical study of medicinal plants in Guji agro-pastoralists, Blue Hora District of Borana Zone, Oromia region, Ethiopia. J Med Plants Stud.

[71] Kebede S, Afework M, Debella A, Ergete W, Makonnen E (2016). Toxicological study of the butanol fractionated root extract of Asparagus africanus lam., on some blood parameter and histopathology of liver and kidney in mice. BMC Res Notes.

[72] Getahun A. Some common medicinal and poisonous plants used in Ethiopian folk medicine. Addis Ababa University, Addis Ababa, Ethiopia; 1976.

[73] Ashenafi A. Current status of ABS implementation in Ethiopia Copenhagen; 2015. http://www.abs-initiativeinfo/fileadmin//media/Events/2015/28-29_January_2015_Copenhagen_Denmark/Ethiopiapdf. Accessed 10 Mar 2017.

[74] Birhanu FM (2010). Challenges and prospects of implementing the access and benefit sharing regime of the Convention on Biological Diversity in Africa: the case of Ethiopia. Int Environ Agreements.

[75] CBD. The Nagoya Protocol on Access and Benefit-sharing. Convention on Biological Diversity. CBD Secretariat. https://www.cbdint/abs. Accessed 14 Mar 2017.

[76] Edwards S, Tadesse M, Hedberg I (1995). Flora of Ethiopia and Eritrea, volume 2, part 2: Canellaceae to Euphorbiaceae.

[77] Edwards S, Tadesse M, Demissew S, Hedberg I (2000). Flora of Ethiopia and Eritrea. Volume 2, part 1. Magnoliaceae to Flacourtiaceae.

[78] Hedberg I (2006). Flora of Ethiopia and Eritrea volume 5.

[79] Hedberg I, Edwards S, Nemomissa S (2003). Flora of Ethiopia and Eritrea. Volume 4, part 1. Apiaceae to Dipsacaceae.

[80] Edwards S, Demissew S, Hedberg I (1997). Flora of Ethiopia and Eritrea. Volume 6. Hydrocharitaceae to Arecaceae.

[81] Friis I. Floristic richness and endemism in the flora of Ethiopia and Eritrea. In: Hedberg I, Friis I, Presson E, editors. Flora of Ethiopia and Eritrea, volume 8 General part and index to volumes 1-7. The National Herbarium, Addis Ababa, Ethiopia, and Department of Systematic Botany, Uppsala, Sweden; 2009.

[82] Hedberg I, Friis I, Edwards S (2004). Flora of Ethiopia and Eritrea. Volume 4, part 2. Asteraceae.

